# Why a positive rheumatoid factor test does not always mean rheumatoid arthritis

**DOI:** 10.1172/JCI210542

**Published:** 2026-10-01

**Authors:** Luc Van Kaer

**Affiliations:** 1 Department of Pathology, Microbiology and Immunology, Vanderbilt University Medical Center, Nashville, Tennessee, USA.; 2 Vanderbilt Institute for Infection, Immunology, and Inflammation, Vanderbilt University Medical Center, Nashville, Tennessee, USA.

## Abstract

Rheumatoid factors (RFs) are autoantibodies directed against the tail region of IgG antibodies and are present in a majority of people with rheumatoid arthritis (RA). RF production is commonly assessed to help diagnose RA, but its performance is limited due to low specificity and sensitivity for reasons that have remained unclear. In this issue of the *JCI*, Hocaoğlu and Sawalha shed light on this matter using extensive phenome-, genome-, transcriptome-, and proteome-wide association analyses of RF-seropositive and -seronegative individuals. They report that RF production was controlled by a genetic signature that included HLA and non-HLA genes and an immune signature reflective of B cell dysregulation but distinct from the genetic and immune signatures associated with RA. These findings explain the limited diagnostic value of RF testing in RA and may help refine the clinical management of RA and development of interventions that prevent or delay disease.

## Rheumatoid factors in rheumatoid arthritis and beyond

Rheumatoid arthritis (RA), which affects 0.5%–1% of the population, is a systemic autoimmune disease that arises in genetically susceptible individuals as a result of environmental factors such as tobacco smoking and certain infections (1–3). Its pathology is characterized by infiltrating inflammatory cells and autoantibodies that mediate persistent inflammation of the joints. Among the hallmark autoantibodies of RA are rheumatoid factors (RFs), which are directed against the fragment crystallizable (Fc) region, that is, the tail end of IgG antibodies (4–7) (Figure 1). Most RFs have the IgM antibody isotype, but they can also exist as IgG and IgA isotypes. These antibodies were first discovered in 1937 (8) and coined RFs because of their association with RA in 1952, and RF serological status was introduced into the classification criteria for RA in 1958. Although RFs continue to serve as serological markers for the diagnosis of RA (9), their clinical value is limited due to low specificity and sensitivity (7, 10, 11).

RFs are found in 60%–80% of patients with RA (4–7). They are thought to contribute to RA clinical disease activity by forming immune complexes that are deposited in the joint lining, resulting in complement system activation, macrophage recruitment, engulfment and destruction of the immune complexes, and production of proinflammatory mediators that instigate tissue damage. In patients with RA, high RF levels in the serum are associated with a poor prognosis and the development of extra-articular manifestations, i.e., damage to organ systems beyond the joints such as the heart and lungs (7), particularly when RFs of the IgA isotype are present, as these are associated with pulmonary lesions.

RFs are not unique to RA, as they can be elevated in a wide range of other conditions (4–7), including other autoimmune diseases (e.g., Sjögren’s syndrome and systemic lupus erythematosus), certain infections (e.g., hepatitis C and EBV infection and bacterial endocarditis), and conditions associated with mucosal inflammation (e.g., periodontal and pulmonary disease). RFs are also found in 3%–10% of healthy individuals, and prevalence increases with age, reaching up to 20%–25% in the elderly population (12). Positive serology for RFs in healthy individuals has limited predictive value for future RA development, unless high titers enriched for non-IgM isotypes are present (7).

Considering these limitations in the diagnostic and prognostic performance of RFs as biomarkers for RA, Hocaoğlu and Sawalha (13) asked whether the genetic and immune signatures associated with RF production mirror those previously identified for RA. Thus, these investigators interrogated large databases containing clinical, biomarker, genetic, and transcriptomic and/or proteomic information from RF-seropositive and -seronegative individuals to profile the genetic and immune landscapes associated with RF serostatus in the context of normal health, RA, and other conditions.

## Distinct genetic signatures

The heritability of RA has been estimated at about 60%, accounting for over half of RA disease susceptibility in the general population (14). The most prominent genetic risk factor is located within the HLA class II region, specifically the *HLA-DRB1* gene (15) (Figure 1). Distinct variants of this gene encode a shared epitope of 5 amino acids that forms a structural motif within the peptide-binding groove to facilitate the presentation of certain self-peptides to autoreactive, CD4-expressing T lymphocytes. In turn, these autoreactive helper T cells facilitate the development of autoantibodies directed against proteins or peptides modified by citrullination — a biological process whereby the amino acid arginine is converted into citrulline (16). Over 100 other non-HLA genetic loci have been implicated in RA susceptibility, including genes that control immune cell activity (e.g., protein tyrosine phosphatase nonreceptor type 22 [*PTPN22*], cytotoxic T lymphocyte antigen 4 [*CTLA4*], and *STAT4*) and protein citrullination (e.g., peptidyl arginine deiminase 4 [*PADI4*]) (1, 3).

Similar to RA, RF production has a strong genetic component. Earlier studies provided evidence that RA and RF production are controlled by distinct genetic loci (17). Hocaoğlu and Sawalha (13) similarly found a strong genetic association with RF-seropositive status. They discovered two susceptibility loci within the extended HLA region, including a locus strongly linked to the *HLA-DQA1* gene. They also found less prominent associations with a variety of non-HLA genes, including genes affecting B cell identity, activation, differentiation, and survival (e.g., paired box 5 [*PAX5*], BTB and CNC homolog 2 [*BACH2*], TNF receptor superfamily member 8 [*TNFRSF8*], and TNF superfamily member 13B [*TNFRSF13B*]), as well as genes influencing Fc receptor biology (e.g., Fc γ receptor 2A [*FCGR2A*]) (Figure 1). Importantly, in sharp contrast to RF-seropositive patients with RA, the asymptomatic RF-seropositive population studied by Hocaoğlu and Sawalha (13) lacked a significant increase in RA genetic risk as compared with the seronegative control group. Thus, these findings provide strong evidence for distinct genetic risk factors associated with the production of RFs compared with RA.

## Distinct immune signatures

The peripheral immune signature of RA reflects the systemic breakdown of immune tolerance mechanisms that unleash tissue-damaging inflammatory pathways. It is characterized by the generation of hallmark autoantibodies such as RFs and anti-citrullinated protein antibodies (ACPAs) (found in 70%–90% of patients with RA, and more specific to RA than RFs) (4, 5), macrophage activation, neutrophil degranulation, activation of naive B and T cells, expansion of effector T cells associated with inflammatory responses and B cell help, elevated inflammatory markers (e.g., erythrocyte sedimentation rate [ESR] and C-reactive protein [CRP]), and production of proinflammatory cytokines (e.g., IL-1β, IL-6, and TNF-α) (18) (Figure 1). These systemic immune alterations in RA were confirmed by Hocaoğlu and Sawalha (13). However, in individuals with asymptomatic RF production, they found a sharply distinct immune signature reflecting mild immune dysregulation, as noted by the induction of features of antibody production, immune complex regulation, IFN-mediated chemokine induction, antigen presentation, and reprogramming of the memory B cell compartment (Figure 1). Most notably, the memory B cells displayed an activated phenotype, and many expressed the death receptor CD95 (also known as FAS). Some of these alterations in the proteomic and transcriptional features of asymptomatic RF-seropositive individuals were consistent with the genetic risk factors for RF production that the investigators had identified.

Furthermore, the immune landscape of RF production Hocaoğlu and Sawalha uncovered was distinct from the peripheral immune phenotypes that previous studies observed in asymptomatic individuals producing ACPAs and in healthy first-degree relatives of patients with RA (19, 20). Interestingly, in asymptomatic RF-seropositive individuals who went on to develop RA, inflammatory and tissue-damaging pathways characteristic of RA appeared to arise at or near disease onset, suggesting that progression from asymptomatic RF production to RA is associated with a shift in immune signatures rather than gradual expansion of the RF signature. Collectively, these findings indicate that RF production and RA display distinct immune signatures and that progression of asymptomatic RF production to RA likely involves a rather quick transition from the mild B cell dysregulation signature of RF production to the more profound tissue-damaging signature of RA, perhaps triggered by environmental factors such as infection (Figure 1).

## Implications and future directions

Hocaoğlu and Sawalha’s findings provide a molecular explanation for the limited value of RF testing in diagnosing RA (21). As RF production and RA are characterized by distinct genetic and immune signatures, the presence of RF antibodies is not directly linked to RA disease. Nevertheless, a gradual rise in RF serum levels, especially RFs with the IgG and IgA isotypes, often precedes the onset or diagnosis of RA (22). Hence, one way RF serology could help in predicting progression to RA would be to test RF-seropositive individuals for sequential elevations in RF isotypes — starting with IgM, and followed by IgG and IgA. Such individuals could then be tested for genetic susceptibility to RA and for the presence of ACPAs. Such testing might permit early intervention to alter the disease trajectory during the so-called “window of opportunity” in RA treatment (23). Similarly, since RFs have been implicated in the pathogenesis of RA, their status can inform disease prognosis, including the development of extra-articular manifestations (7). In this context, the phenome-wide association analyses performed by Hocaoğlu and Sawalha (13) confirmed an association between stable, asymptomatic RF production and respiratory diseases such as hypersensitivity pneumonitis, a finding consistent with the proposed mucosal origin of RF production and its progression to RA (24).

RF testing also holds promise for predicting the RA therapeutic response, as high RF titers have been associated with resistance to treatment with disease-modifying antirheumatic drugs, including conventional synthetic drugs such as methotrexate, and certain biological agents such as Fc receptor–containing anti–TNF-α antibodies that can be bound by RFs and targeted for degradation (25). Finally, the RF-associated immune signatures identified by Hocaoğlu and Sawalha (13) could be harnessed for therapeutic purposes. The molecular pathways associated with these immune signatures could serve as the foundation for developing precision therapeutics, utilizing existing drugs or novel compounds, in efforts to prevent or delay RF production and progression to RA or to modify the disease trajectory.

In conclusion, the study by Hocaoğlu and Sawalha (13) elegantly illustrates how multiomics data in clinical databases can be leveraged to better understand the genetic and molecular underpinnings of disease.

## Conflict of interest

The author has declared that no conflict of interest exists.

## Funding support

This work is the result of NIH funding, in whole or in part, and is subject to the NIH Public Access Policy. Through acceptance of this federal funding, the NIH has been given a right to make the work publicly available in PubMed Central. This work was supported in part by the following:

- NIH grant R01AI139046 (to LVK).

## Figures and Tables

**Figure 1:**
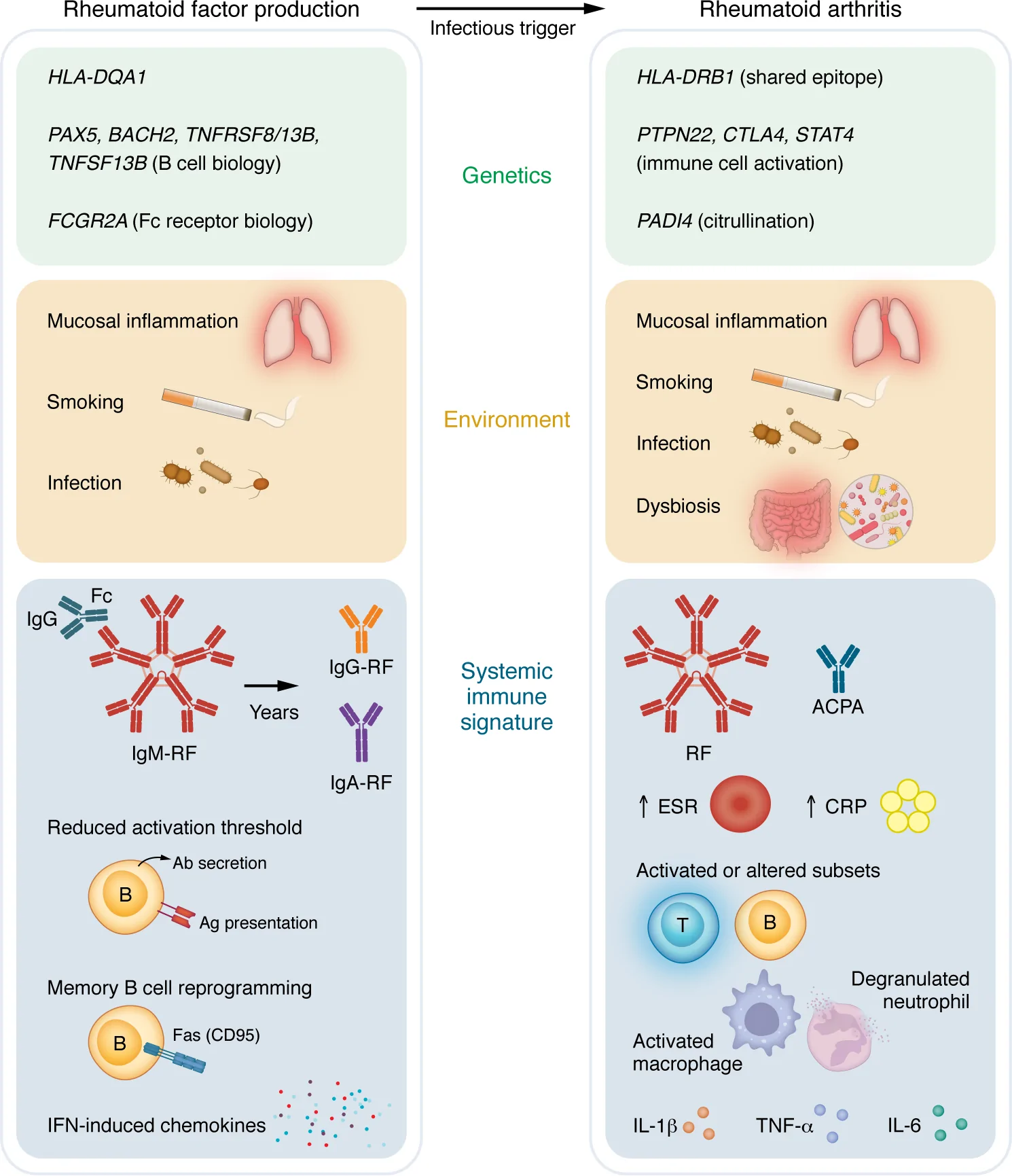
Genetic, environmental, and immune factors associated with asymptomatic RF production compared with RA. Hocaoğlu and Sawalha (13) compared the genetic and systemic immune signatures of RF production in asymptomatic individuals (left) with the established genetic and peripheral immune signatures of patients with RA (right). In addition to differences in the genetic loci associated with either group, the study determined that RF production in genetically susceptible individuals may be associated with environmental factors such as smoking and certain infections that mediate mucosal inflammation. Over time, RF levels may rise and include non-IgM isotypes. In individuals who are also genetically susceptible to RA development, environmental factors such as infections may trigger progression toward RA and rapid induction of the systemic RA immune signature, which commonly includes production of ACPAs.

## Version 1 10/01/2026

Electronic publication
